# Farmers’ Strategies to Climate Change and Urbanization: Potential of Ecosystem-Based Adaptation in Rural Chengdu, Southwest China

**DOI:** 10.3390/ijerph19020952

**Published:** 2022-01-15

**Authors:** Bo Zhong, Shuang Wu, Geng Sun, Ning Wu

**Affiliations:** 1 China-Croatia “Belt and Road” Joint Laboratory on Biodiversity and Ecosystem Services, CAS Key Laboratory of Mountain Ecological Restoration and Bioresource Utilization & Ecological Restoration and Biodiversity Conservation Key Laboratory of Sichuan Province, Chengdu Institute of Biology, Chinese Academy of Sciences, Chengdu 610041, China; zhongbo@cib.ac.cn (B.Z.); sungeng@cib.ac.cn (G.S.); 2 University of Chinese Academy of Sciences, Beijing 100049, China; 3 College of Built Environments, University of Washington, Seattle, WA 98195, USA

**Keywords:** ecosystem-based adaptation (EbA), Chengdu Plain, climate change, urbanization, agricultural system, traditional knowledge or practice, functioning ecosystem, agro-biodiversity

## Abstract

Ecosystem-based adaptation (EbA) is emerging as a cost-effective approach for helping people adapt to climate and non-climate changes. Nowadays, climate change and urbanization have affected agricultural systems, but it is not clear how rural communities have responded or adapted to those changes. Here, we chose two typical villages in the Chengdu Plain, southwest China, through sociological surveys on 90 local farmers with a semi-structured questionnaire, participatory observation, geospatial analysis of land use and land cover, and a literature review, to explore the local people’s perception of changes or disturbances and their adaptation strategies from the perspective of EbA. The results showed that climate change and urbanization had impacted agricultural systems dramatically in the last 40 years. In two case-study sites, climate change and urbanization were perceived by most local farmers as the main drivers impacting on agricultural production, but various resource-use models containing abundant traditional knowledge or practices as well as modern tools, such as information communication technology (ICT), were applied to adapt to these changes. Moreover, culture service through the adaptive decoration of rural landscapes is becoming a new perspective for implementing an EbA strategy. Finally, our findings highlighted the potential value of an EbA strategy for sustaining urban-rural integrated development and enhancing the resilience of agricultural systems.

## 1. Introduction

Sustainable agriculture is the foundation of social and economic development and plays an important role in national food security [1]. In recent decades, with the continuous advancement of industrialization and urbanization around the world [2], a large number of agricultural lands and human populations have been transferred or imported to nearby cities [3]. Considering widely-discussed climate change [4] and rising urban demand for agricultural products [5], the pressures on agricultural systems have increased continuously, especially for those located in peri-urban or suburban areas [6]. Agricultural hard infrastructure, such as flood barriers, engineered irrigation systems and facilities-based intensive agriculture, was believed to be able to effectively cope with climate change and significantly increase agricultural outputs for meeting the demands for agricultural products during the process of urbanization [7]. Although the investment in agricultural hard infrastructures has contributed to the agricultural adaptation to changes, they were considered likely to bring about negative influences, since these engineered solutions could end up working against nature, particularly when they are aimed at constraining ecologically important processes, and that finally would result in several uncertainties regarding ecosystem functioning in the future [8].

Ecosystem-based adaptation (EbA), which was defined as integrating the use of biodiversity and ecosystem services into an overall strategy for helping people to adapt to changes [9,10,11,12], is becoming a prioritizing option globally and being promoted by many international organizations, such as the International Union for Conservation of Nature (IUCN), Global Environment Facility (GEF), and the United Nations Environment Program (UNEP) [13,14]. Unlike other engineer-based solutions, EbA shows a more inclusive solution which could harness the capacity of nature to buffer human communities against adverse impacts through the sustainable delivery of ecosystems services [12,15]. Moreover, it reflects human effort to tackle global challenges with ecosystem-based solutions, i.e., aspiring after a more harmonious relationship with nature, a traditional but newly emerging wisdom of humankind. The main body of EbA involves multiple levels, including national and regional governments, local communities, private companies, non-governmental organizations (NGOs), and even individual farmers [14,16]. Therefore, there are a number of case studies for EbA application and management since it has been demonstrated to create multiple environmental, socioeconomic, and cultural co-benefits [10,15,17].

Initially, several EbA studies were conducted in poor or high-altitude rural areas, such as Tegucigalpa in Honduras [18], Bash-Kaiyndy in Kyrgyzstan [11], Vu Quang district in Vietnam [19] and Troms and Finnmark County in Norway [12], and the proposed EbA strategies were also proven to effectively improve the ability of local farmers to cope with climate change. Recently, growing attention have also been paid to EbA strategies at an urban level for addressing climate change challenges [20]. Lafortezza et al., (2013) emphasized that enhancing green infrastructures in urban areas could reduce the heat island effect and the related health risks to local inhabitants [21]. Subsequently, the construction of urban parks, green roofs, tree belts or artificial wetlands, were proposed to help mitigate the adverse effects of heat, flood, or drought events in urban areas [20,22,23]. While considering urban–rural systems as a continuum, however, agricultural landscapes in rural areas, as hotspots of biodiversity providing various ecosystem services to urban landscapes, has not yet been properly understood or has even been overlooked, even though it is affected directly by the knock-on effects of climate change and urbanization. Based on the EbA perspective, therefore, exploring the adaptation strategies in rural areas would be critical for sustaining agricultural development and urbanization in coping with various ongoing changes with a nature-based solution.

The Chengdu Plain is one of the greatest examples in the world to show rich agro-biodiversity and environmental heterogeneity supporting the long-term resilience of agricultural systems and the sustainability of human wellbeing [24,25,26]. As a fast-growing metropolitan area, Chengdu is not only the capital of the Sichuan Province in southwest China, but it is also the most important economic, commercial, cultural and transportation hub in western China [3]. During the past decades, however, natural and socioeconomic driving forces such as climate change and urbanization have caused great impacts on the agricultural system which have further hindered the regional sustainable development of the Chengdu Plain, a place called “The Land of Heavenly Abundance” (*tian fu zhi guo* in Chinese) in China [27,28,29]. Therefore, a comprehensive understanding on EbA in rural Chengdu is essential to ensure the agricultural landscape is being well planned and developed sustainably, which could also be beneficial for the redevelopment of this region based on biodiversity conservation and ecological restoration, such as the ongoing national initiatives of “Rural Revitalization” and “Constructing Park City”.

In this study, we conducted a field survey in two typical villages (called “*cun*” in Chinese) of the Chengdu Plain, combined with spatial analysis and a literature review, to learn the impacts of climate change and urbanization on the agricultural system, as well as adaptation strategies adopted by the local agrarian society. The aims of this study include: (1) documenting climate change and urbanization in the past 40 years; (2) analyzing farmers’ perception of changes and related EbA knowledge or practices; and (3) exploring how to improve the efficiency of EbA to achieve regional sustainable development.

## 2. Materials and Methods

### 2.1. Case Study Sites

**Figure 1:**
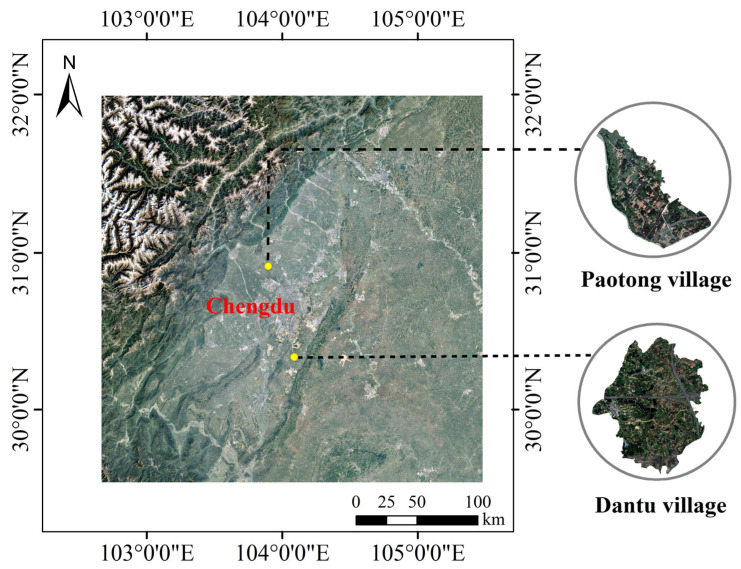
Location of case study sites in Chengdu Plain.

The Chengdu Plain is an alluvial basin located between 103°–104°42′ E longitude and 29°31′–31°50′ N latitude, about 110 km long from north to south and 80 km wide from west to east, with an area about 14,000 square kilometers and a total population of over 20 million (Figure 1). Flowing down from the Dujiangyan Irrigation System in the northwest, a world cultural heritage site, the network of irrigation canals has supported the agricultural prosperity of this plain over the last 2000 years and it is still irrigating a vast area of about 6700 km^2^ [26,30]. Taking Chengdu as an example, in order to better understand the EbA in rural areas, research was carried out in two typical villages, i.e., Paotong Village, Pidu District, about 26 km to the northwest of the urban center, and Dantu Village, Shuangliu District, about 30 km to the south (Figure 1).

**Figure 2:**
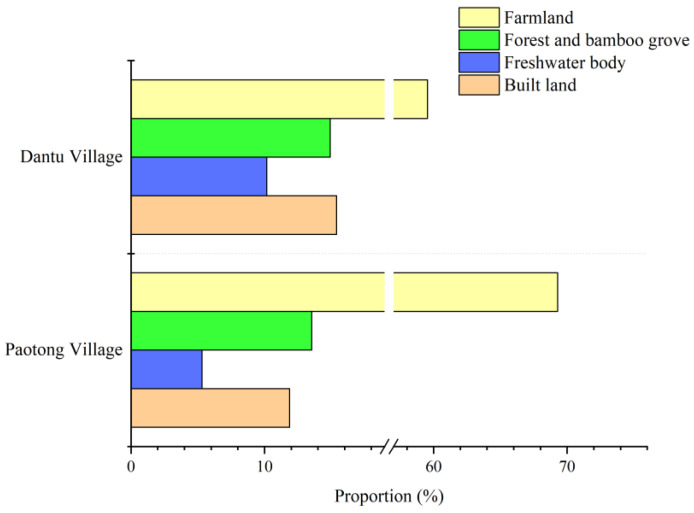
The Proportion of land cover in two case-study sites.

**Figure 3:**
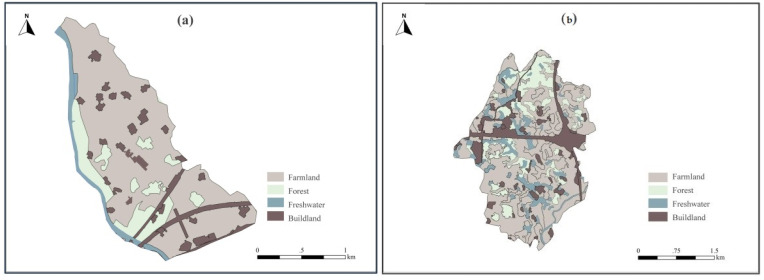
Land use of Paotong Village (**a**) and Dantu Village (**b**).

Paotong Village, with an area of 1.87 km^2^ and a population of 2290 in 2018, is a typical agricultural area where local people traditionally engaged in crop cultivation. Based on the Google Earth image in November 2018, the spatial tools of ArcGIS 10.2 were used to visually interpret the land cover in the area, which were further verified through a field survey. It could be found that farmlands make up 69.29% of land cover, followed with forests and bamboo groves (13.53%), built lands (11.87%, including settlements and roads) and freshwater bodies (5.31%, including canals and ponds) (Figure 2 and Figure 3a). This area belongs to the oldest part of the ancient Dujiangyan Irrigation System watershed with very flat topography and fertile soil and is dominated by the *linpan* landscape [26], an integrated ecosystem complex including farmer houses surrounded by tree and bamboo groves, farmlands (mainly paddy fields) and freshwater bodies (mainly canals, ditches or ponds).

Dantu Village is another type of agricultural village with an area of 6.45 km^2^ and a rural population of 3457 from 1072 households in 2018. The landscape of Dantu Village is mainly composed of farmlands (59.54%) and built lands (15.39%), followed by forests (14.91%) and freshwater bodies (10.16%) (Figure 2 and Figure 3b). Given the elevation of 500–550 m, the topography of Dantu Village is gently hilly. Forests (e.g., native woodlands and plantations) can be found at the ‘up-slopes’ of gentle hills, and the freshwater bodies (mainly canals and water ponds) are normally distributed nearby to farming fields, most of which are located alongside relatively low-lying lands and surrounded by tree/bamboo groves. Thus, the agricultural landscape presents heterogeneous patches along with topographical variation.

### 2.2. Methods and Data Collection

Climatic data including precipitation and temperature from 1978 to 2018 were taken from the Chengdu Meteorological Bureau (Pidu Station), and urbanization data such as urbanization rate, gross domestic product (GDP) per capita, agricultural products and recreation consumption were extracted from the *Chengdu Statistical Yearbook* [31]. The case study in two villages was carried out during the field surveys in 2019 and 2020. The questionnaire was semi-structured around key topics (Table 1), which focused on the perceptions and opinions related to the sense of climate changes, urbanization and ecosystem-based approaches for adaptation. Through the introduction of village committee (called “*cun wei hui*” in Chinese) staff, we firstly interviewed 5 local farmers from different families in two villages, respectively, all of whom have good experience in agricultural activities, and required them to review the questionnaire to ensure that the prepared questions were understandable and appropriate. Then, the revised questionnaire including ten categories of questions was used for in-depth surveys, which were conducted with 40 respondents in the Paotong Village and 50 respondents in the Dantu Village, respectively. The gender balance and age structure of the interviewees were considered. For example, women make up 20% and 26% in the two groups, respectively. Considering the importance of experience with engaging in agriculture, the farmers with age over 35 were the main interviewing targets, most of whom had lived in the village for over 10 years, being 92.5% and 96%, respectively, in the two case-study sites. The basic information of respondents is shown in Table 2 below. Moreover, in the Paotong Village and Dantu Village, respectively, two and three open discussions with knowledgeable elders and village leaders (village committee staff) were held to learn the local governance system, social institutions and policies related to adaptation and agricultural development. Additionally, traditional knowledge or practices related to biodiversity use were also documented through a field survey and literature review. The method of participatory observation was used when the authors stayed with local people who were difficult or reluctant to provide details, such as how much was earned from agricultural product trading. In this way some interviewed results could also be tested and verified.

### 2.3. Climate Change and Urbanization in Chengdu

**Figure 4:**
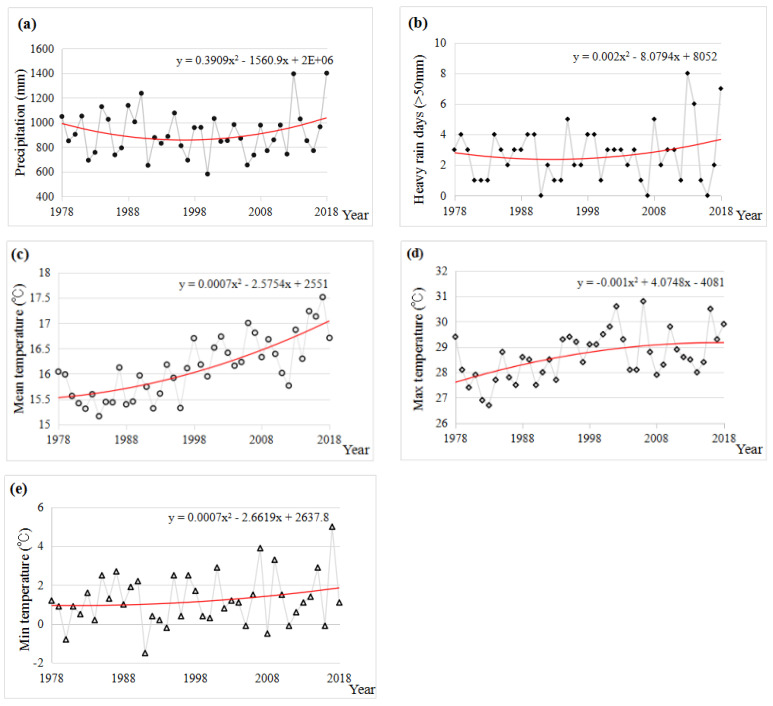
Annual precipitation (**a**), heavy rain days (**b**), mean temperature (**c**), max temperature (**d**) and min. temperature (**e**) during 1978–2018 in the Chengdu Plain.

Currently, increasing climate change has become a great concern to governments and the public in China [2], especially annual mean precipitation, heavy rain days (the total amount of precipitation in 24 h exceeding 50 mm), annual mean temperature, and maximum and minimum temperatures [32]. According to the meteorological monitoring data, the annual mean precipitation and heavy rain days showed a similar change in the last 40 years in Chengdu, especially with the increasing trends observed since 1998 (Figure 4a,b). In addition, the annual mean temperature showed an obvious upward trend from 1978 to 2018, and a marginal upward occurrence in maximum and minimum of mean temperatures (Figure 4c–e).

**Figure 5:**
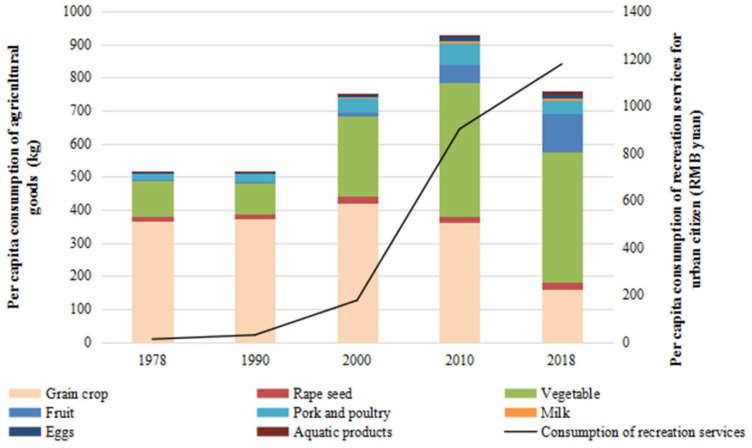
Consumption of agricultural goods and services per capita from 1978 to 2018 in Chengdu metropolitan area.

China’s accelerated development during the last 40 years has resulted in rapid urbanization with unprecedented large-scale population movements. Regarding the Chengdu metropolitan area, the urbanization rate in 1978 was only 22.26%, but in 2000 it had reached 53.72%, and then quickly reached 73.12% in 2018 (Table 3). Therewith, the built-up area in Chengdu increased 16.35 times from 57 km^2^ in 1978 to 932 km^2^ in 2018 to meet the demand for living space of an increasingly urban population. The demographic and land-use urbanization caused by the rural-urban migration led to the reduction of a rural population engaged in agriculture from 2.41 million in 1978 to 1.49 million in 2018, a decrease of about 38.17%. At the same time, the area of arable land for crop cultivation decreased by about 25.87%, from 9973 km^2^ in 1978 to 7393 km^2^ in 2018, which further drove the changes of the agricultural system as well as the traditional agrarian society. Moreover, with the rapid rise of GDP per capita in Chengdu from 1978 to 2018 (Table 3), the consumption of grain crops per capita showed a downward trend, while the consumption of vegetable and fruits per capita, as well as recreation services per capita (only for urban citizens) had increased significantly after the year 2000 (Figure 5), which finally led to a livelihood diversification from purely agricultural production to a mixture of horticulture, rural tourism, agricultural product processing and/or commerce.

## 3. Results

### 3.1. Farmers’ Perception about Changes

#### 3.1.1. Climate Change

**Figure 6:**
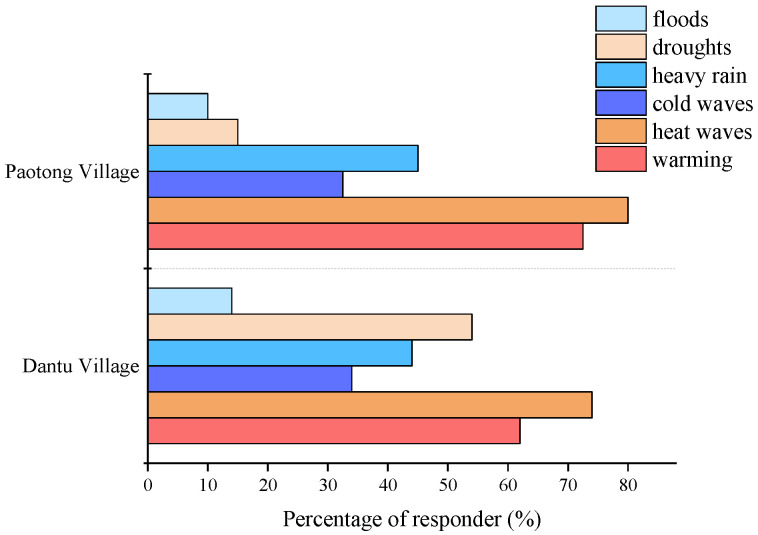
Respondents’ perception to climate change and weather extremes.

According to the field survey in two case-study villages (Figure 6), most of the respondents were aware of warming effects (72.5% in Paotong Village and 62% in Dantu Village) which were consistent with the officially monitored data (Figure 4). The results showed that more than 30% of respondents in the two villages recognized that weather extremes (such as heat wave, cold wave, and heavy rain) had occurred more frequently in the past 20 years. Briefly speaking, heat wave was noticed by 80% and 74% of respondents in the Paotong Village and Dantu Village, respectively, as the most frequent influence in the last 20 years. Furthermore, although 45% and 44% of respondents in the Paotong Village and Dantu Village, respectively, mentioned the influence of heavy rain in summer, the impact of floods was considered on the contrary to be not so serious (only 10% in Paotong Village and 14% in Dantu Village, respectively), for which fine irrigation systems were appreciated. Regarding drought impacts, the responses were different. In Dantu Village, there were more respondents (54%) who identified drought occurring more frequently than that in Paotong Village (15%). The reason for this is perhaps due to the differences of topography and irrigation condition in the two villages. The recognition of cold wave conditions was similar in the two sites, i.e., 32.5% in Paotong Village and 34% Dantu Village, respectively. Generally, the awareness of local people of weather extremes was varied in different places, which were not always consistent with the monitoring data. In the Chengdu Plain, the climatic conditions are similar in such a short distance (between the two case-study villages), regardless of temperature and precipitation, but people’s feelings of the impacts of them were changeable between the two locations.

**Figure 7:**
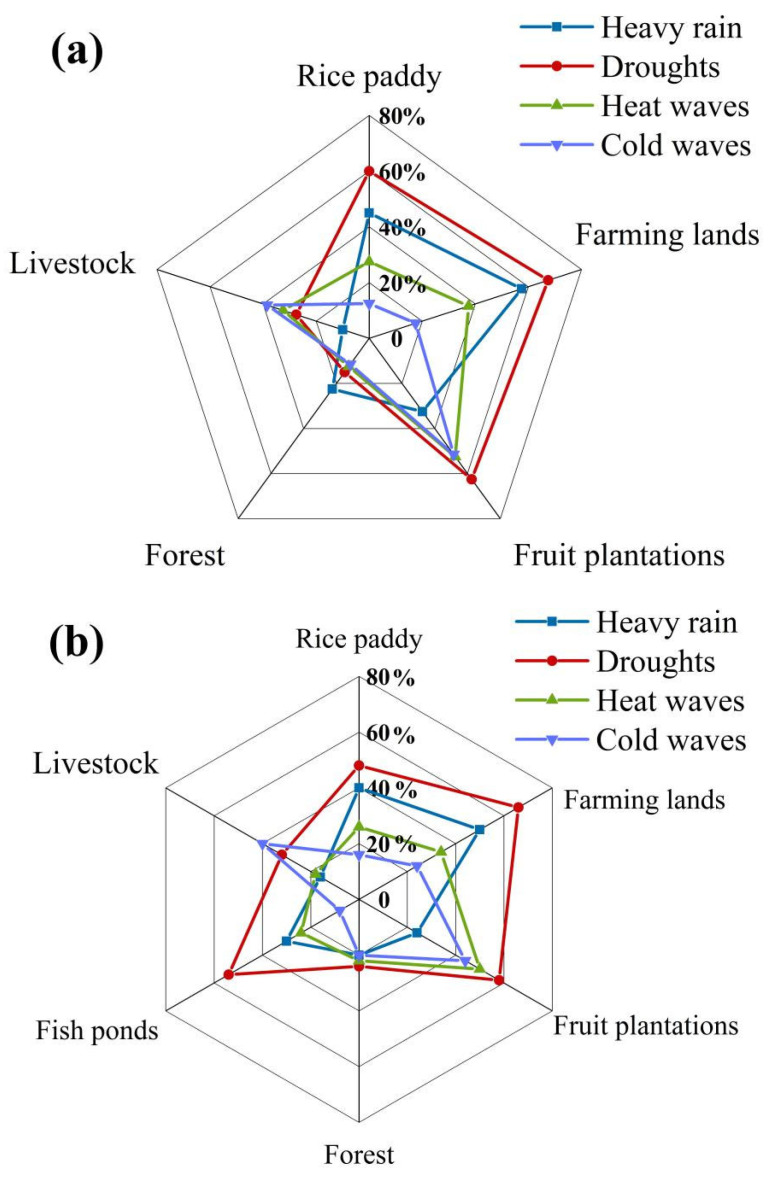
The perceived impacts of weather extremes on agricultural systems in Paotong (**a**) and Dantu (**b**) villages.

As to the impacts of climate change on different agricultural systems (Figure 7), there were 67.5% of respondents in the Paotong Village and 66% in the Dantu Village who perceived that farmland was more susceptible to drought, followed by fruit plantations, rice paddies and fish-ponds (only in Dantu Village). Heavy rain was recognized as the second factor which most affected farmlands, rice paddies and fruit plantations. More than 50% of respondents (57.5% in Paotong Village and 50% in Dantu Village, respectively) complained of the reduction of agricultural harvests due to heavy rains in summer. Moreover, fruit plantations and farming fields were considered to be more sensitive to heat waves than other systems in both the Paotong Village and Dantu Village. Interestingly, although heat waves occurred more frequently than cold waves (Figure 6), the respondents showed more attention to the cold waves. This is because a cold wave could result in not only a decline in crop production but also an expansion of epidemic diseases and even the death of domesticated animals, especially poultry and pigs.

#### 3.1.2. Climate Change

**Figure 8:**
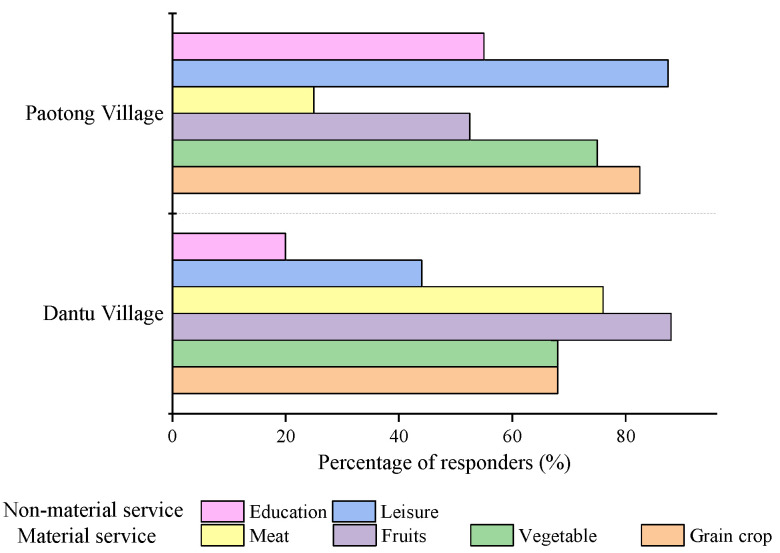
The perceived goods and services delivered to the urban market in Paotong Village and Dantu Village.

With the rapid urbanization process in Chengdu, increasing opportunities for income generation in urban areas have driven the labor out-migration from rural areas (Table 3). At present, mostly elderly people and women still engage in agriculture in the case-study villages. The increasing demands of urban citizens on diverse material and non-material ecosystem services have further inevitably led to the livelihood diversification of the agrarian society. In addition to traditional crop cultivation, for instance, about 87.5% and 72% of respondents’ households in Paotong Village and Dantu Village, respectively, engaged partly in non-farming activities in 2019. In Paotong Village, over 50% of respondents recognized that grain crops, vegetables and fruits were the important products provided to urban markets in the last 20 years, while meat supply (e.g., chicken, pork and duck) was less mentioned (Figure 8). As for non-material services, cultural services (e.g., leisure and education) delivered by the agricultural landscape were mentioned by over 55% of respondents, which has increased very quickly in the last five to ten years.

For the Dantu Village, material services delivered to the urban market, such as grain crops, vegetables, fruits and meat, were mentioned by 68% of respondents, especially fruits and meat, which were recognized by 88% and 76% of respondents, respectively (Figure 8). For the non-material services (i.e., recreation, leisure and education), less than 50% of respondents mentioned them during the survey. The difference of delivered goods and services reflects the different strategies of livelihood diversification in the two villages to adapt to urbanization.

### 3.2. EbA Strategies in Coping with Changes

#### 3.2.1. EbA Strategies for Climate Change

**Figure 9:**
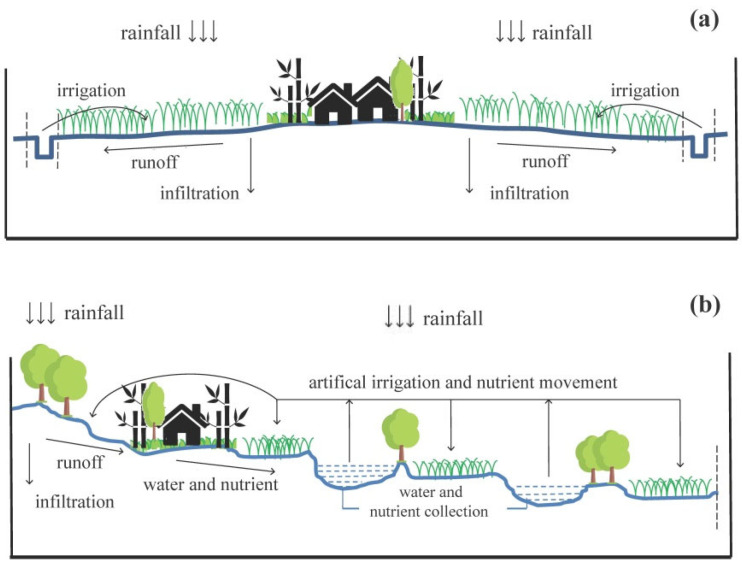
Conceptual modes of agricultural landscape of Paotong (**a**) and Dantu (**b**) villages.

In the two case-study villages, all respondents acknowledged that a functioning ecosystem was the foundation for coping with climate change. As to weather extremes such as heavy rain, drought and heat waves, freshwater bodies, including hydraulic systems were identified by over 80% of interviewees in both villages as the most important ecosystem, which could effectively alleviate the adverse impacts (Table 4). In Paotong Village, for instance, the freshwater network (i.e., different canals or ditches) linked with the Dujiangyan Irrigation System could not only drain rainwater-runoff efficiently from rice paddies with heavy rain coming, but it could also irrigate those fields while a drought or heat wave was striking (Figure 9a).

In Dantu Village, the complicated agricultural system integrating woodland, farmland and freshwater bodies (e.g., water pond and fish farm) into a topography-based vertical model, could enhance the ecological resilience to weather extremes efficiently (Figure 9b). Within this complicated system, for example, broad-leaved or pine forests distributed on the upper slopes or tops of gentle hills could protect rice paddies or farming fields from rainwater-runoff and soil erosion, and discharge groundwater stored in forest soil slowly into water ponds down-slope during heavy rain. On the other hand, in the case of drought and heat wave striking, farmers could irrigate their farming fields or fruit plantations with water stored in numerous surrounding ponds.

**Figure 10:**
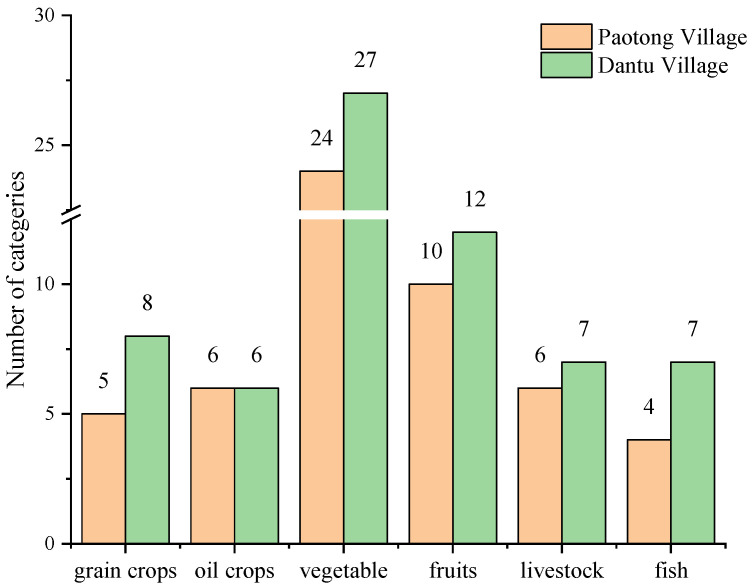
The agricultural biodiversity in two case-study sites.

Sustainable use of agro-biodiversity is fundamental to EbA. In the Paotong Village there are about 64 species of domesticated plants and animals, including 5 kinds of grain crops (i.e., rice, wheat, maize, broom corn and potato), 6 kinds of oil crops (i.e., rape, peanut and 4 kinds of beans), 24 kinds of vegetables (e.g., tomato, cabbage, chives, pepper, eggplant, chili pepper, pumpkin, cucumber, radish, and cauliflower), 11 kinds of fruits (e.g., grape, plum, loquat, pear, peach, mandarin, strawberry, melon, orange, persimmon, and cherry), 6 kinds of domesticated animals (duck, chicken, goose, rabbit, sheep, and cow) and 4 kinds of fish (e.g., *Ctenopharyngodon idella*, *Mylopharyngodon piceus*, and 2 varieties of *Hypophthalmichthys molitrix*) (Figure 10). In the Dantu village, due to the heterogeneous environment, there are more diverse species/varieties/breeds of grain crops, vegetables, fruits, livestock, poultry and fishes. For example, there are two varieties of potato (purple potato and jicama), three varieties of vegetable (garlic, sweet pepper and okra), two varieties of fruit (grape and watermelon), one breed of domesticated animal (pig), and three species of fish (*Aristichthys nobilis*, *Pelteobagrus fulvidraco* and koi fish), more than those of Paotong Village, respectively.

Rational use of agro-biodiversity has also strengthened farmers’ capacity to cope with weather extremes (Figure 6 and Figure 7). Considering the impacts of heavy rain on agriculture in the Paotong Village and Dantu Village, farmers usually choose special rice varieties (e.g., with low-height and high-production properties) to ensure a stable yield. Moreover, with increasingly frequent heavy rainfalls in spring and early summer, which can lead wheat to being flattened, the farmers preferred to cultivate rape crops instead of wheat in winter (Table 4). Meanwhile, with drought and heat wave suddenly occurring in summer, farmers usually cultivate alternative crops (e.g., sweet potato) flexibly in order to offset the rice loss. In Paotong Village, due to the plentiful water resources and convenient irrigation conditions, local farmers like to cultivate some vegetables with a water preference, such as water spinach, chives, cucumber and tomatoes, etc. On the contrary, considering the relative lack of water on the up-slopes, in the Dantu village crops with drought resistance were planted in the terraced fields. In the case of a cold wave occurring during winter time, the farmers in both villages would re-plant cold-resistant crops immediately, such as radishes, carrots, onions, lettuce, kale, etc.

In addition to crop cultivation, some traditional knowledge or practices can also be helpful for alleviating the impacts of weather extremes. For reducing soil moisture evaporation with drought and heat waves prevailing, farmers in the Dantu Village would collect tree leaves and litter from forests or bamboo groves to cover the grounds of their farming fields and orchards. Agricultural litter (such as straw or leaves) were also used (as a mat) in poultry or pig pens to enhance their cold resistance. Burning agricultural litter in fruit orchards during a prevailing cold wave was a traditional practice to cope with a severe drop in temperature. With 3–5 bonfires per acre in one plantation, air temperature could be increased by about 1–2 °C. For maintaining enough temperature and water of the soil, watering a field (inundated by water) before planting crops was also a useful way to maintain soil condition, which now has been adopted by the local people in Dantu Village to reduce insect pests during strawberry cultivation (also for reducing the use of pesticides).

#### 3.2.2. EbA Strategies for Urbanization

With the increasing demands for diverse goods and services from rural areas during the process of urbanization, farmers have been aware of material and non-material (e.g., recreation) services that the agricultural landscape could supply. Due to the different topographical features and ecosystem types, the identified services in the two villages were different, which related to their respective EbA strategies. In the Paotong Village, owing to the fertile soil and convenient hydraulic system, grain crops, vegetables and horticultural plants were cultivated mainly for urban demands. At the same time, the well-developed transportation of this village was convenient for urban people to spend their weekends or holidays, and thus numerous *linpan* (farmers’ settlements) were planned and decorated as new recreation spaces for rural tourism, providing homestay, tea garden and restaurant services. In the Dantu Village, the cultivation of traditional grain crops had declined. Rice was mainly for self-consumption, but maize was used for wholesale in the market. The rape crop had replaced wheat, becoming the main crop during winter time. Many varieties of fresh fruits were introduced in the last 10 years, and interplanted with vegetables in orchards or home gardens.

The lack of sufficient labor due to out-migration had also driven the transformation of agricultural activities and then livelihoods. The cultivation of grain crops is labor-intensive and some farmers can now order agricultural services through smart phone applications (APP), such as for rice transplanting and harvesting, for example, a professional team might help older people to complete tasks that they are unable to complete themselves at a certain expense (according to how big their paddy field is). In the Paotong Village, some nursery gardens were developed to provide seedlings, decorating plants or flowers to urban markets. In the Dantu Village, water ponds were used for multiple purposes, such as fish farming, angling game and scenery observation. The blooming flowers of fruits and rape crops in spring attracted urban tourists, who brought additional income for the local population in addition to the agricultural products themselves. For the purpose of rural tourism development, traditional practices such as pottery making and bamboo weaving were being revitalized, which had become new markets for sightseeing programs. The livelihood of local households had, thus, been diversified for adapting to the ongoing influx of urban culture.

#### 3.2.3. EbA Strategies for Agricultural Management

Agricultural management is important for sustaining the vitality of agricultural systems. A diversified agricultural system is supposed to generate positive feedback effects for crops and domesticated animals [33], contribute to an increase of agricultural outputs in a limited area, and reduce labor or fertilizer costs. Generally speaking, a diversified agricultural system illustrates a tempo-spatial arrangement of agricultural activities. Spatially, diversification includes the interplanting of different crops and the combination of cultivation and animal breeding within a given area. In the Dantu Village, for instance, fruit trees such as orange, mandarin, pear and loquat were normally planted on contour lines where seasonal vegetables were intercropped under their canopy, in particular various beans or peas which could fertilize the fields through nitrogen fixation. Cultivation mixed with poultry breeding under fruit trees could also be commonly found. Temporally, diversification usually refers to the rotation of dissimilar types of crops in the same field. In the Paotong Village and Dantu Village, for example, farmers used to mix different varieties of rice, and the annual output was satisfied by most of the farmers in comparison to a single variety. A rotation system could usually sustain continuous production and enhance the resilience during weather extremes or a market failure striking.

Efficient recycling of nutrients is essential for a functioning agricultural system, which is one of the advantages of an integrated ecosystem complex. In Dantu Village, for example, the nutrients of forest soils on the up-slopes are released with the runoff slowly and nourish the farming fields located on the down-slopes (Figure 9b). The sludge at the bottom of fish ponds was also dug out and distributed on the farming fields nearby during water discharge (mostly in winter). Due to the rich composition of fish excrements, the sludge could be considered a good fertilizer for crop cultivation, which further saved the cost of purchasing chemical fertilizer. In both the Paotong Village and Dantu Village, farmers made compost (with vegetable residues and animal manure) for accelerating nutrient activation. Although electronic appliances have been popularized in rural areas, the dependency on firewood has decreased, with local farmers still preferring to collect some fuels (e.g., leaves and branches) from forests, plantations and their gardens nearby. The purpose is to reduce the cost of electricity use (for cooking) and to recycle residues because the burnt ashes were used traditionally as potash fertilizer.

An agricultural system cannot be operated well without socioeconomic and technological interventions, in particular while facing the pressures of rapid urbanization (Table 3). In the two villages, several farmers’ associations of specific products such as vegetable, fish or fruit were organized under the support of a village committee or enterprises. For example, in August 2014, the agricultural association for vegetable production was established in Paotong Village in which more than 80 households were involved. During our field investigation, over 70% of interviewees admitted that agricultural associations played an important role in coordinating agriculture production at the village level. By providing new seeds or varieties, exploring potential markets, and building the capacity of their members, these associations empowered local farmers when adapting to new challenges or uncertainties. In Dantu Village, for instance, after many fish ponds were developed, a fishery association was organized by those households who operated the fish farms. Initially, the association was only responsible for coordinating fish breeding and marketing issues among members. Later, it was developed as a platform for knowledge and practice sharing, capacity building, and new technology piloting. The principal members of the fishery association progressively grew from 8 in 2007 to 408 in 2018, including farmers from Dantu Village as well as some from neighboring villages.

With the assistance of the farmers’ associations, new technologies were introduced and applied in agricultural production and local governance. In the Dantu Village, a new facility for in-time feeding of fish and a machine for cleaning the sludge of fish ponds were automatically introduced by the fishery association in 2013. With these technologies, labor costs were saved and the quality of the aquatic products increased to the required standard of the urban market. In 2014, a fish-pond management system based on information communication technology (ICT) was introduced, with which farmers could monitor water quality and control fish feeding at any time through their smartphones.

In Paotong Village, a fine-grained governance tool based on ICT, called “grid management system” was applied, which presented opportunities to sustain and scale up the collection of data necessary for regulating agricultural production and enabling local governance. Grid managers used specially designed cellphones with a geolocation-enabled APP to collect and report the issues that they find on their patrol routes. The responsibilities of grid managers included: coordinating social and administrative services, reporting emergencies and responding, providing aids to vulnerable groups (e.g., elder, disabled), and promoting policies and regulations. The information reported by these local managers would be gathered and analyzed quickly by local nets or the county’s information center, where acting staff could advise on tackling these issues efficiently. Although the system was not specially designed for ecosystem-based adaptation purposes, new technologies have shown opportunities for enhancing the efficiency and effectiveness of agricultural management. For example, it could enable a large scale of knowledge or information sharing and an efficient way for addressing environmental concerns (e.g., water pollution), further strengthening the local capacity for implementing EbA strategies.

### 3.3. Farmers’ Perception about the Importance of EbA Strategies

As mentioned above, a functioning ecosystem, agro-biodiversity, traditional knowledge and self-organization were identified as crucial roles in EbA strategies. With the average importance over four points perceived by more than 75% of respondents, a functioning ecosystem and agro-biodiversity were popularly recognized in the two case-study sites as the fundamental base for adapting to climate change and urbanization (Table 5); however, self-organization, such as farmers’ associations or cooperation, was perceived as a more effective approach in their adaptation to urbanization (4.15 points in Paotong Village and 4.28 points in Dantu Village) than climate change (3.5 points in Paotong Village and 3.8 points in Dantu Village). Although traditional knowledge or practices such as rotation, inter-cultivation and manure preparation was very important, farmers still prefer to use chemical fertilizer due to its high ‘effectiveness’, and thus they were not rated too highly in either case-study villages. In the Dantu Village, perhaps due to the diverse environmental conditions, about 40% of respondents still recognized traditional knowledge as ‘important’ to climate change so that the mean point reached 3.28, which was higher than that in Paotong Village (2.95). As to the adaptation to urbanization, the ‘importance’ of traditional knowledge was not acknowledged enough by respondents, showing a conflict between traditional and modern approaches adopted by local communities in their adaptation.

## 4. Discussion

With the global movement of sustainable urban-rural development and the world-wide consensus about “think global, act local” adaptation strategy [34], in this study we took two typical villages in the Chengdu Plain to explore how local communities adapt to climate change and urbanization from the perspective of an EbA, which undoubtedly would be beneficial to the enhancement of EbA and helpful to the implementation of sustainable development in the future.

### 4.1. Comparison of EbA with Other Adaptations

All adaptation strategies aim to cope with adverse external changes, providing multiple benefits and striving to transform to sustainable development pathways. For example, ecological engineering (EE), catchment systems engineering (CSE) [35] and green infrastructure (GI) [20,23,36] represent targeted adaptation at the governmental level to solve several specific problems (e.g., floods, heat waves and air pollution). At the level of individuals, farmers manage their agricultural system to cope with or adapt to changes by combining modern technology (drought-tolerant varieties) and traditional knowledge (cropping pattern, fertilizing and composting) [37]. Although the adaptation mentioned above strategies have produced certain effects, they are still lacking synergetic impacts due to ignoring holistic considerations on the agricultural systems and weakening enforcement of climate-responsive policies for agriculture. On the contrary, an EbA adopts a systemic approach that endorses a more inclusive and participatory approach, highlighting the importance of considering different interests and conflicts [16]. EbA involves not only collective action among national and regional governments, local communities, private companies, NGOs and other stakeholders, but also contains different individuals for addressing different pressures (including climate change and urbanization) on ecosystem services and for managing ecosystems to increase regional resilience to climate and non-climate changes [14,16]. Therefore, EbA could provide more social benefits to a larger and broader area [15], and has gained attention in policies as a flexible, low-risk and low-cost approach to adapting to changes [11,20,23,38]. In this study, it was found that traditional practices and modern technologies have been used by local people to increase their capacity of EbA. Depending on local agro-biodiversity and ecosystem services, farmers could manage their agricultural system flexibly to adapt to climate change and urbanization, such as reducing agricultural production losses under extreme weather conditions and transferring dependency on provision services to cultural services.

### 4.2. Agro-Biodiversity Conservation Being Fundamental to EbA

Biodiversity could strengthen the resilience of agricultural systems, and help smallholders to diversify their livelihoods, contributing to the mitigation and adaptation strategies that could further reduce the sensitivity of rural households to external changes [39]. In two case-study villages, it was found that the cultivation of various grain crops, vegetables and fruits, not only improved the abilities to cope with climate change and adapt to ongoing urbanization, but also contributed to the income generation of local farmers. Compared with the cultivation of single-variety rice, the mixed model with a number of rice varieties has been demonstrated to be effective in controlling diseases or pests [40] and enhancing the resilience of farming systems [41]. According to the third national investigation of crop resources in China [42], there are plentiful varieties of cultivated crops including 484 of rice, 103 of wheat, 115 of maize, 16 of sorghum, 152 of soybean, 17 of barely and 96 of rape plants in the Chengdu Plain. This rich agro-biodiversity is thus critical to maintaining the stability of the whole agricultural system while external pressures (e.g., weather extremes) are occurring and providing farmers with diverse choices for adaptation.

Moreover,, the agricultural landscape in the Chengdu Plain is actually a complicated ecosystem complex that assembles forests, wetlands and farmlands in one place where diverse species, including wild and domesticated species, live together. For example, it was reported that 181 species of plants belonging to 146 genera and 76 families were found in the *linpan* landscape of Chengdu Plain which forms diverse vegetation types [43,44] and provide ideal habitats for birds and other wildlife. The mixture of different species in the agricultural landscape thus could effectively ensure food safety through continuing the harvest of diverse products under climate variations [19], and this kind of ecosystem complex can take advantage of the mutual benefits between species and their complementary use of resources. Liu et al., (2013) demonstrated that a diversified cultivation model, such as legumes planted between fruit trees, could promote fruit yield through nitrogen supply by the nitrogen fixation capacity of legumes [45]. Beekeeping is another case showing this kind of mutual benefit between different species, which could be found popularly at our case-study sites. It was recognized that pollination is necessary for plant reproduction [46], such as grain, vegetables, and fruits as well as plentiful wild plants, which has a significant correlation with crop yields [47].

Conservation, however, and the use of biodiversity sometimes also incurs new challenges. For example, in our case-study sites, wheat, a traditional grain crop, was replaced by maize and rape crops. Some farmers complained that wheat is susceptible to diseases, less tolerant to extreme weather (e.g., increasing rainfall in spring and early summer), and is easily eaten by birds which increased rapidly due to recent forest enrichment, all of which might lead to a decline in production. In the Dantu Village, it was noted that the better the ecological conditions became, the more the loss of grain crops due to a bird increase would be, which in fact indicates a new interactive relationship among biological species.

### 4.3. Well-Structured Rural Landscape Consolidating EbA

A heterogeneous rural landscape with rich agro-biodiversity was considered to be essential for the formation and maintenance of ecosystem services for the lives and livelihood of both urban and rural people. Forests distributed in rural landscapes could alleviate flood impacts caused by storms, maintain the soil and fertility, regulate air temperature and maintain biodiversity [26]. An appropriate location of those forests could maximize multiple benefits. In the Chengdu Plain, taking the *linpan* as an example, the function of regulating micro-climate by trees or bamboo groves around farmers’ settlements, i.e., warming in winter but cooling in summer [48], has been recognized popularly by both local people and scholars [24]. These bamboo and trees in the *linpan* are not only important habitats for birds that could control insects or pests in the surrounding farming fields, but they also provide fuel woods, construction material and even foods directly to local communities [26]. Moreover, it was also demonstrated that an agricultural system combined with trees could significantly improve the ability of coping with climate change and generating cash income in comparison to non-tree agricultural systems [49].

Moreover, a well-structured agricultural landscape usually ensures EbA strategies gain “twofold results with half effort.” As a typical model of a traditional agricultural system in China, the Hani Terraced Paddy in Yunnan province, with its vertical interval of forest, village, rice terrace and wetland, shows the perfect harmonious relationship between humans and nature, which was recognized by FAO and UNESCO as a world heritage site [45]. The Hani model illustrates how an EbA practice, “hydrological fertilization”, distributes soil nutrients stored in mountain forests to rice terraces below with the fine-turning drainage system of rainwater or runoff [50]. In the Dantu Village, the topography-based agricultural system with three belts (forest, farmland, and freshwater body) on gentle slopes, being consistent with the Hani model, could be regarded as its “epitome”. Forest enrichment after the 1980s in Dantu Village improved the adaptive capacity of local communities with the increasing regulation services delivered by forests such as a cycling water resource, mitigating weather extremes and maintaining soil fertility, which further contributed to the sustainable production of the agricultural system (e.g., farming field, orchard and fish-pond). The freshwater bodies, including water ponds and canals, also play an important role in controlling flush floods while heavy rain is coming and supplying necessary irrigation to fields during the dry season. The farmlands finally benefit from the runoff nutrients of the forests. Therefore, a diverse and well-structured agricultural landscape with different ecosystems co-existing together could present the synergetic effects in delivering comprehensive ecosystem services to human wellbeing locally and regionally.

### 4.4. Good Governance Sustaining EbA

As to the implementation of EbA strategies at the local level, some potential obstacles could undermine the effectiveness, such as (i) limited resources-time, people and financial [23,51], (ii) insufficient knowledge-underlying impact and adapting strategies of external changes [52,53], and (iii) limited communication and cooperation between local farmers (stakeholders) and governments [23,51,53,54]; however, a “down-to-earth” institution organized by local farmers can properly deal with problems such as limited resources and knowledge as well as communication with local farmers (stakeholders) and governments. As for integrating and coordinating socioeconomic resources as well as different specialized agricultural knowledge, Fontanari (2017) thought local institutions (e.g., cooperatives or associations) play a critical role in societal responses to climate and non-climate changes [55]. Moreover, these institutions at the grass-roots level which are usually recognized and supported by local governments, occupy an essential position that governments are unable to perform [56], such as crop varieties acquisition, good practice sharing, market exploring, as well as new technology piloting.

In the last 40 years, there have been considerable changes in the agrarian society in China. The rural reform in the 1980s led to the change of land tenure, rural institutions and governance system [2]. The collective activities of agricultural production were transformed into being household-based, which provided the opportunities for making decisions by farmers themselves. Finally, the governance system of resource management was decentralized into a diverse community or household-based institutions such as farmers’ associations or administrative village committees. In both the Paotong and Dantu villages, more than 70% of interviewees recognized that agricultural cooperatives played an important role in agricultural development and resilient society building. The self-organization of farmers provided incentives and opportunities for adapting to changes and undoubtedly supported the successful implementation of EbA practices. Akamani (2016) also emphasized that the self-organization of farmers could help to enhance awareness, generate interest, and create opportunities for enhancing the transition toward an EbA [57].

Recently, with the progress of science and technology, farmers’ associations began to integrate local knowledge with new technology such as ICT-based tools to enhance their abilities of adaptation. The application of an intelligent management system (e.g., Internet of Things) in Dantu village provided a convenient way for monitoring fish ponds online, thereby enhancing the effectiveness and efficiency of resource management. The newly emerging ICT-enabled governance tool, i.e., “Grid Management System” (GMS), gives local communities an opportunity to correct what they see as a potentially maladaptive trend in agricultural development, strengthen feedback loops between different scales of decision making, and achieve a balance between local choices and regional integration. The combination of traditional knowledge with emerging technologies would surely enable local governance and consolidate EbA actions, which would be beneficial to the long-term resilience of agricultural systems [58].

## 5. Conclusions

Presently, EbA is being popularized and adopted at national, regional and local levels. Based on an EbA perspective, in this study we conducted a survey on the impacts of climate change and urbanization and local EbA knowledge or practices in two villages of the Chengdu Plain. The results showed that climate change and urbanization have impacted agricultural systems dramatically in the last 40 years and suggested the critical role of a functioning ecosystem and biodiversity for the effective implementation of an EbA strategy. As to the local adaptations to climate change and urbanization, local communities have accumulated abundant traditional knowledge and developed various resource-use models based on local biodiversity and ecological heterogeneity to cope with these changes. Now, it has been widely recognized that most of the adaptations have proved to be derived from local agricultural ecosystems with traditional knowledge, practices and lifestyles handed down by local farmers for hundreds or thousands of years. As an agricultural landscape, the *linpan* landscape in the Chengdu Plain is a carrier of abundant EbA strategies, which has supported the agricultural prosperity in history; however, farmers’ perception on the importance of traditional knowledge has declined in the case-study villages due to the out-migration of the younger generation through the process of urbanization. In order to promote the implementation of EbA strategies in the future, preservation and inclusive use of traditional knowledge or practices should be thus paid more attention by policy makers and development agencies.

Several agricultural associations or organizations were proved to be effective in promoting local agricultural development and adaptation capacity, while the establishment and operation of agricultural associations also face many difficulties, such as inadequate funding, lack of social awareness, technical guidance and good leadership. It was found in the two case-study villages that farmers’ associations, primarily established on a voluntary basis, could avoid disorderly or unfair competition among members (for villagers), reduce external transaction costs by expanding the scale of the organization, and efficiently disseminate new techniques or market information to local communities. Therefore, local governments should provide financial, technical and even political supports to these associations, promote them to collaborate with enterprises as well as consultative agencies, and give them discourse power in decision making related to agricultural development, all of which could be greatly helpful for improving the efficiency of EbA implementation. The mutual flows of ecosystem services from rural areas to urban areas or vice versa, and connecting natural ecosystems with human societies, could not only contribute to the EbA operation for agricultural systems, but also strengthen EbA capacity in both urban and rural society, synergizing the supply of ecosystem services to a metropolitan area in its entirety. Recently, in order to uncover the interactive mechanisms of the urban–rural continuum in delivering ecosystem services, comprehensive studies have been applied to analyze the agricultural landscape that is not visible to either social or natural scientists who study human and natural systems separately. With the development of 3S technologies (GIS, GPS and remote sensing), cloud computation, and ICT-based technologies, spatial optimization and landscape architecture of the urban–rural continuum based on the flow of ecosystem services, has also emerged as a hot topic; however, due to the limitation of enough data, we still could not quantify the flow of ecosystem services in the *linpan* landscape, although it should be an important topic in the future. Moreover, integrating the culture service of agricultural landscapes into urban planning is becoming a new perspective for the implementation of EbA strategies, but less attention have been paid to cultural services than to provision services, which inevitably undermines the effectiveness of integration. Despite this, the potentially great value of these new approaches or perspectives combined with traditional knowledge and practices for strengthening local EbA capacity should be highlighted, which will undoubtedly contribute to the long-term resilience of agricultural systems and regional sustainable development.

## Figures and Tables

**Table 1:** Categories of semi-structured questionnaire around key topics.

Number	Key Questions
1	Do you think it has been getting warmer in the last 20 years? (yes/no)
2	Do you think there are more extreme heat waves in summer compared with the last 20 years? (yes/no)
3	Do you think there are more droughts compared with the last 20 years? (yes/no)
4	Do you think there has been an increasing heavy rainfall over the last 20 years? (yes/no)
5	Do you think the number of flood disasters has increased in the last 20 years? (yes/no)
6	Do you think the number of cold waves has increased in the last 20 years? (yes/no)
7	Which climate changes (such as heat waves, drought, heavy rain, flood, and cold waves) have severely impacted agricultural production or agricultural systems (rice paddy, farming land, fruit plantation, forest, or livestock)? How do you adapt to the impacts based on agro-biodiversity (species, varieties or breeds) and/or agricultural system?
8	Considering natural resources and agricultural products in your village, what services do you think are important for urban demands (a list of material supply and non-material supply)?
9	How can you sustain the deliverables including goods and services to the urban market? What are the advantages and/or disadvantages for your household?
10	According to the above replies, please assess the importance of EbA practices which you have adopted in the last few years (1–5 point; 1: not important; 2: not so important; 3: general importance; 4: important; and 5: extremely important).

Note: As to the changes, the time scale was 20 years in the questionnaire, but it was an open question if respondents could answer to their feelings for the past 40 years.

**Table 2:** The structure of surveyed farmers in two case-study villages.

	Respondent Structure	Composition (Number of Persons)
Paotong Village	gender	men (32), women (8)
	age	under 35 (2), 35–45 (7), 45–55 (12), over 55 (19)
	residence time	under 10(3), 10–20(4), 20–30(8), over 30 (25)
Dantu Village	gender	men (37), women (13)
	age	under 35 (3), 35–45 (8), 45–55 (13), over 55 (26)
	residence time	under 10 (2), 10–20 (4), 20–30 (13), over 30 (31)

Note: “Residence time” means how many years a person has lived in this village.

**Table 3:** The process of urbanization in Chengdu from 1978 to 2018.

	1978	1990	2000	2010	2018
Urbanization rate (%)	22.26	27.30	53.72	65.76	73.12
Built-up area (km^2^)	57	277	3874	455	932
Rural population engaged in agriculture (10^4^ person)	241.36	305.13	244.09	152.65	148.58
Sown area of crops (km^2^)	9973.41	9909.44	9880.93	6398.87	7393.14
GDP per capita (RMB Yuan)	565	2123	11,471	41,253	94,782

**Table 4:** EbA strategies to cope with weather extremes in the two villages.

		Paotong Village		Dantu Village	
Weather extremes	Mainly impacted system	Functioning ecosystem based adaptation	Agro- biodiversity based adaptation	Functioning ecosystem based adaptation	Agro- biodiversity based adaptation
Heavy rain	Farmland and fruit plantation	Using hydraulic system (canals) for drainage	Cultivating flatten resistant varieties (e.g., short straw rice varieties) or crops (e.g., replacing wheat with rape crop); cultivating water preference vegetables (e.g., water spinach, chives, cucumber and tomatoes)	Storing and discharging rainwater with forests; using water ponds for collecting water	Cultivating flatten resistant varieties (e.g., short straw rice varieties) or crops (e.g., replacing wheat with rape crop)
Drought	Rice paddy, farmland, and fruit plantation	Using hydraulic system for irrigation	Cultivating drought resistant crops (e.g., peas, broad bean and soybean)	Using water ponds for irrigation; covering ground surface with tree leaves or litter	Cultivating drought resistant crops (e.g., sweet potatoes, maize, peas, broad bean and soybeans)
Heat wave	Farmland and fruit plantation	Using hydraulic system for irrigation	Cultivating heat resistant crops (e.g., maize)	Using water ponds for irrigation; covering ground surface with tree leaves or litter	Cultivating heat resistant crops (e.g., sweet potatoes and maize)
Cold wave	Farmland, fruit plantation, poultry and pig breeding	Spreading straw onto the ground of animal pens	Cultivating cold resistant crops (e.g., radishes, carrots, onions, lettuce, kale etc.)	Watering field before planting crops; using bonfire to increase air temperature; spreading straw onto the ground of animal pens	Cultivating cold resistant crops (e.g., radishes, carrots, onions, lettuce, cabbage, kale, peas and beans)

**Table 5:** Farmers’ perception of the importance of EbA strategies.

EbA Strategies	Paotong Village		Dantu Village	
	Climate Change (Percentage of ‘>4 Points’)	Urbanization (Percentage of ‘>4 Points’)	Climate Change (Percentage of ‘>4 Points’)	Urbanization (Percentage of ‘>4 Points’)
functioning ecosystem	4.05 (85%)	4 (80%)	4.24 (88%)	4.12 (84%)
agro-biodiversity	4 (80%)	4.05 (75%)	4.44 (96%)	4.08 (80%)
self-organization	3.5 (40%)	4.15 (85%)	3.8 (68%)	4.28 (92%)
traditional knowledge	2.95 (20%)	2.9 (25%)	3.28 (40%)	3.04 (28%)
functioning ecosystem	4.05 (85%)	4 (80%)	4.24 (88%)	4.12 (84%)

Note: The importance of EbA strategies was ranked with 1–5 point; 1: not important; 2: not so important; 3: general importance; 4: important; and 5: extremely important.

## Data Availability

Not applicable.
